# A gamma-ray spectrometer based on MAPD-3NM-2 and LaBr_3_(ce) and LSO scintillators for hydrogen detection on planetary surfaces

**DOI:** 10.1038/s41598-025-85845-y

**Published:** 2025-01-22

**Authors:** F. Ahmadov, A. Sadigov, Yu. Yu. Bacherikov, O. Okhrimenko, K. Isayev, M. Holik, T. Slavicek, F. Mamedov, G. Ahmadov, A. Mammadli, R. Akbarov, J. Nagiyev, D. Berikov, S. Nuruyev, Z. Sadygov, Yu. Shitov, S. I. Lyubchyk, S. B. Lyubchyk

**Affiliations:** 1 Institute of Radiation Problems- Ministry of Science and Education, B.Vahabzade Str. 9, Baku, AZ1143 Azerbaijan; 2 Department of Nuclear Research of IDDA, BakuShamakhy HW 20 km, Gobusett. ofAbsheron dist., Baku, AZ 0100 Azerbaijan; 3 Azerbaijan University of Architecture and Construction, AynaSultanova St.5, Baku, AZ1073 Azerbaijan; 4 V. Lashkaryov Institute of Semiconductor Physics NAS of Ukraine, 45 Nauky Ave., Kyiv, 03028 Ukraine; 5 V. I. Vernadsky Institute of General and Inorganic Chemistry NAS of Ukraine, Academician Palladin Ave., 32/34, Kyiv, 03142 Ukraine; 6 Faculty of Electrical Engineering - University of West Bohemia in Pilsen, Univerzitní 26, Pilsen, 306 14 Czech Republic; 7 Institute of Experimental and Applied Physics-CzechTechnicalUniversity in Prague, Husova 240/5, Prague, 110 00 Czech Republic; 8 Khazar University, 41 Mahsati Str., Baku, AZ1096 Azerbaijan; 9 The Institute of Nuclear Physics, Ibragimova 1, Almaty, 050032 Kazakhstan; 10 REQUIMTE, NOVA School of Science and Technology, University New of Lisbon, Caparica, 2829-516 Portugal; 11 DeepTechLab, RCM2+. UniversidadeLusófona, Campo Grande, 376, Lisboa, 1749-024 Portugal

**Keywords:** Energy science and technology, Physics

## Abstract

The presented work is dedicated to the detection of hydrogen, using detectors based on a MAPD (Micropixel Avalanche Photodiode) array based on new MAPD-3NM-2 type photodiodes and two different scintillators (LaBr_3_(Ce) and LSO(Ce)). The physical parameters of the MAPD photodiode used in the study and the intrinsic background of the scintillators were investigated. For the 2.223 MeV energy gamma-ray indicating the presence of hydrogen, the energy resolution was 6.89% with the MAPD array and LSO scintillator-based detector, and the number of events corresponding to this energy was 4817. With the MAPD array and LaBr_3_(Ce) scintillator, the energy resolution for the 2.223 MeV gamma-ray was 3.55%, and the number of events corresponding to this energy was 3868. The LSO scintillator-based detector allowed for the detection of 24.5% more 2.223 MeV energy gamma-rays compared to the LaBr_3_(Ce) scintillator. For the 2.223 MeV gamma-ray associated with hydrogen, the energy resolution with the LaBr_3_(Ce) scintillator was 48.5% better than with the LSO scintillator. The lower energy resolution compared to the LSO is due to the higher light output of LaBr_3_(Ce). The obtained results experimentally demonstrate that it is possible to obtain information about the presence of hydrogen in the target using both detectors.

## Introduction

Currently, neutron-induced compositional analyses are widely applied in the study of planets^1–9^ and asteroids^10–12^. Neutron sources such as neutron generators, neutrons produced by cosmic radiation, and radioactive sources (e.g., AmBe, CmBe, Cf) are widely used^13^. The energies of neutrons emitted from some neutron generators are monoenergetic, at 2.5 MeV and 14.1 MeV^13,14^, while the energy of neutrons emitted from radioactive sources ranges from 0 to 11 MeV^14^. Additionally, the energy of neutrons generated on planetary surfaces due to cosmic radiation is in the range of 1–20 MeV^3,6^. In these studies, which investigate the composition of targets through the impact of fast neutrons, information about the target’s composition is obtained by detecting gamma rays emitted when neutrons are scattered multiple times with the target nuclei, losing energy and becoming thermal neutrons, or when neutrons are absorbed, and the excited nucleus returns to its ground state. Based on the energy and count of these recorded events, information about the elements within the target can be obtained^6^. It should be noted that not all detected gamma rays are necessarily produced by neutron interactions; they could also originate from the decay products of isotopes such as ^40^K, ^238^U, and ^232^Th, which contribute to the natural background of the planet, or from gamma rays generated by cosmic radiation^3,4,15,16^.

A particularly interesting aspect of these studies, conducted on planetary surfaces and near-surface depths, is the investigation of the presence of water and frozen water using neutrons^17–21^. These investigations identify water by detecting hydrogen. In regions with water, the high amount of hydrogen causes fast neutrons to lose energy through multiple elastic collisions, converting to thermal neutrons and reflecting to the surface. The number of these scattered neutrons, recorded by a detector, provides information about the composition of the rock^2^. A significant increase in the number of recorded thermal neutrons in the studied areas indicates the presence of water (or hydrogen-rich material) in those regions. In most cases, thermal neutrons are captured by hydrogen isotopes, resulting in the emission of 2.223 MeV gamma rays (n^1^ + H^1^_1_ → H^2^_1_ + γ (2.223 MeV))^6^. Each nucleus has its characteristic gamma lines. These emitted gamma rays, when detected, confirm the presence of hydrogen compounds (water or other substances). For this purpose, semiconductor and scintillator detectors are widely used to detect gamma rays^9,12,13,15^. Detector systems used for space research require several special conditions, such as compact size, weak temperature dependence, low operating voltage, resistance to vibration, low power consumption, operation over a wide energy range (0.1–10 MeV), radiation resistance, and cost-effectiveness^6,13,15^. Semiconductor detectors are currently considered the most optimal in terms of energy resolution. This advantage is related to the low energy required to create an electron-hole pair (Si-3.6 eV, Ge-2.98 eV) and the small Fano factor (0.1–0.13)^13^. However, despite these advantages, issues such as high cost, high operating voltage, low radiation resistance, operation at low temperatures, small active thickness, and high energy consumption create certain challenges for the application of semiconductor detectors in space research^22,23^. Therefore, the use of scintillator detectors is considered more optimal for space research. Scintillator detectors consist of two main parts: a scintillator (which converts the energy of ionizing radiation into visible scintillation photons) and a photodetector (which converts scintillation photons into electrical signals)^13^. In scintillator detectors, the detection of ionizing radiation does not occur directly through the creation of electron-hole pairs as in semiconductor detectors. Instead, scintillators sensitive to the type of ionizing radiation are selected. For example, LiI and stilbene for neutrons, ZnS for alpha particles, and NaI, CsI, LSO, BGO, and LaBr_3_(Ce) for gamma rays are used as scintillators^13^. When ionizing radiation interacts with the scintillator, it spends its energy on creating electron-hole pairs. Depending on the type of scintillator, this energy ranges between 10 and 20 eV^13,24–26^. Electrons that have transitioned to the conduction band due to the energy lost by ionizing radiation move to activator centers (which are created by the activator added to the scintillator), and when the charge carriers transition from this energy level to the valence band, scintillation photons are emitted, with energy corresponding to the energy levels of the bands. The number of these scintillation photons emitted by the scintillators varies between 8000 and 100,000 photons per 1 MeV of absorbed energy, depending on the type of scintillator^13,24–26^. Of course, the number of emitted photons can vary depending on the type of ionizing particle. The scintillation photons observed at the output of the scintillator are then transmitted to the photodetector. A photodetector is used to convert the generated scintillation photons into an electrical signal^27–33^. Photodetectors such as PMT and SiPM are of particular interest. SiPM-based photodetectors, which have been improved over the past few years, are considered more suitable for space research compared to PMTs due to their compact size, low operating voltage, resistance to vibration, low energy consumption, radiation resistance, and other parameters^27–35^.

SiPMs were first developed in 1990 and are currently considered sensitive photodetectors to single photons, with improved parameters^35–41^. These photodetectors operate in Geiger mode, with internal gain varying between 10^4^ and 10^7^, achieved through impact ionization^36^. Scintillation detectors based on surface-structured SiPMs have been extensively studied by many researchers^42–50^. In these detectors, increasing the pixel density of the diode leads to a significant decrease in photon detection efficiency (PDE). Consequently, this type of structure is not conducive to developing SiPMs with both high pixel densities (10000 pixels/mm²) and high PDE (~ 25%)^31,34^. As a result, the energy resolution and linear operating range of scintillation detectors using surface-structured SiPMs degrade markedly. To address this issue, the use of a deep buried pixel structure has been proposed as a more effective alternative^31^. SiPMs with deep buried pixel structures currently offer pixel densities ranging from 5,000 to 40,000 pixels/mm² and PDE values between 12% and 35%^31^.More detailed information about the types and operating mechanisms of SiPMs with deep buried pixel structure can be found in works^35–41^.It is for this reason that the improvement of SiPM with deep buried pixel structure and the development of detectors based on them are very relevant. Additionally this structure has high radiation hardness to compare with surface structure^51^. Currently, SiPM with deep buried pixel structure can be considered the most optimal structure for the development of scintillation detectors with excellent linearity over a wide range of energy and good energy resolution^27,31^.

In the presented work, the physical properties of new MAPD-3NM-2 type photodiodes, the 16-element array prepared based on these diodes, and the detection of point gamma sources and gamma rays resulting from the interaction of neutrons with PET using LaBr_3_(Ce) and LSO scintillators were investigated.

## Experimental circuit and results

### Parameters of the MAPD photodiode

The MAPD-3NM-2 type of SiPM used in the experiment are considered the latest version of deep-pixel photodiodes produced by Zecotek Photonics Inc. The thicknesses of the 1st and 2nd epitaxial layers of this structure were 10 and 2 micrometers, respectively. The pixel size was 12 micrometers, and the distance between them was 3 micrometers. The size of the MAPD-3NM-2 photodiode was 3.7 mm by 3.7 mm. A single MAPD-3NM-2 type photodiode contained a total pixel density of 66,500 pixels.

**Fig. 1:**
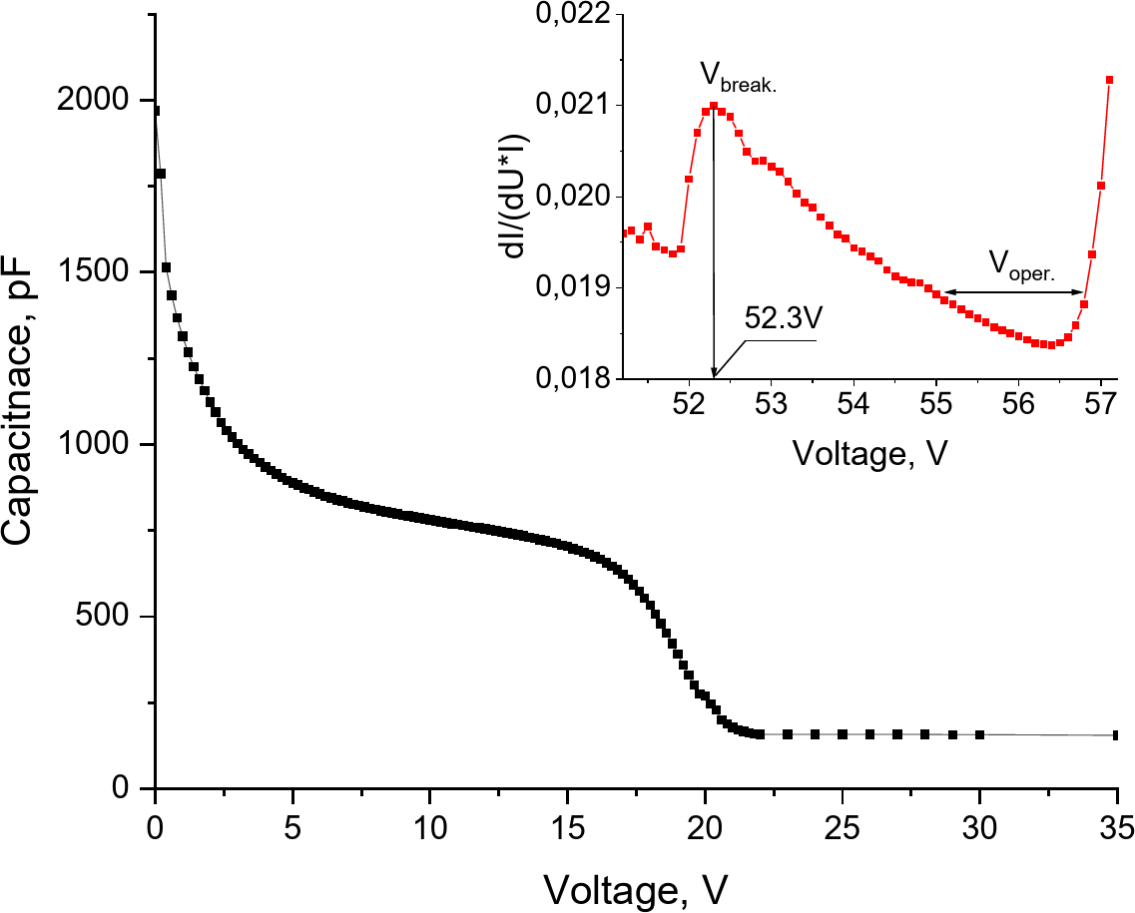
The dependence of the capacitance and the dI/(dU*I) ratio of the MAPD-3NM-2type photodiode on the voltage.

Figure 1 shows the I-V characteristics of the MAPD-3NM-2 type photodiode, measured with a Keithley-6487 device. As seen from the dependence of the differential dI/(dU*I) on the applied voltage, the breakdown voltage of the MAPD-3NM-2 type photodiode was 52.20 ± 0.027 V, and the operating voltage ranged from 54 to 56.3 V.

Figure 1 also shows the C-V characteristics of the MAPD-3NM-2 photodiode, measured with the E7-20 device. To determine the capacitance of the MAPD photodiode using the E7-20 device, a signal frequency of 1 MHz and an amplitude of 40 mV were used. A voltage of 22 V was required to fully deplete the active volume of the photodiode with the charge region. The capacitance of the photodiode was 157 pF.

**Fig. 2:**
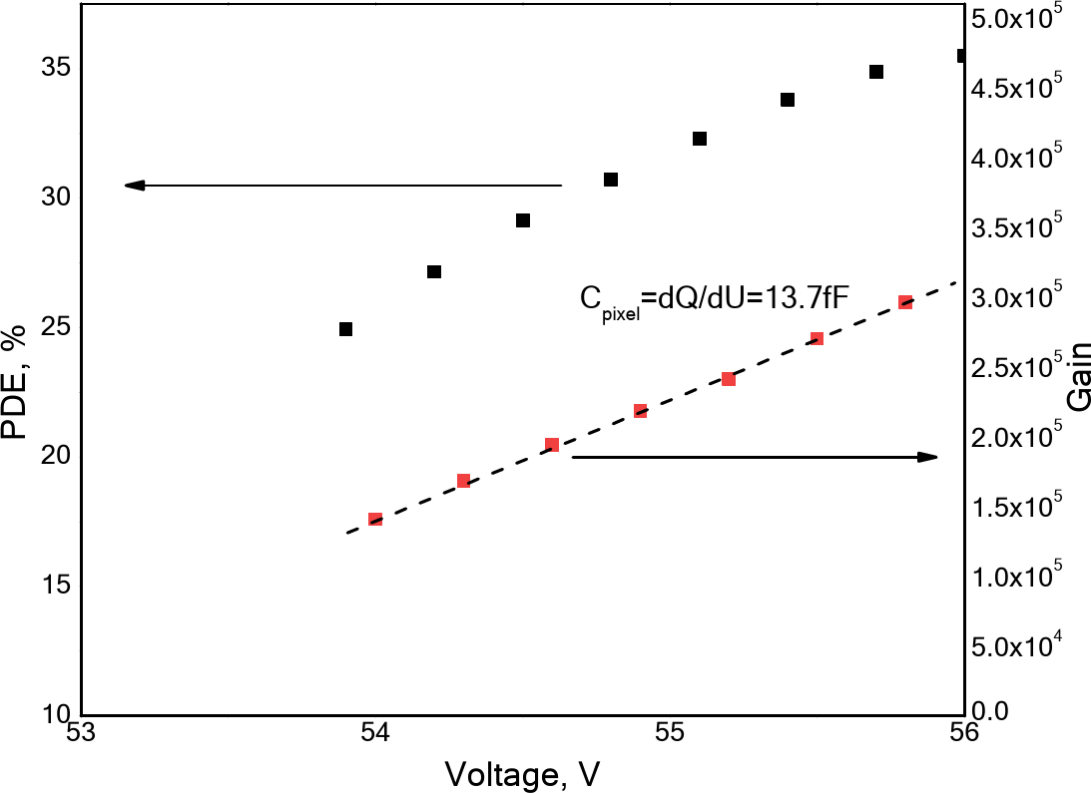
The dependence of gain and PDE on the voltage for the MAPD-3NM-2 type photodiode.

Figure 2 shows the dependence of the gain of the MAPD-3NM-2 type photodiode on the voltage. The gain was calculated based on the amplification of each pixel. More detailed information about the determination of the gain and PDE can be found in works^32,35^. The charge corresponding to one photoelectron was 38.7 fC(55.2 V). Using the voltage dependence of the gain, the breakdown voltage was determined to be 52.3 ± 0.06 V. The gain of the MAPD-3NM-2 type photodiode varied between 1.2 and 3 × 10^5^. This value fully corresponds to the previously determined value. The capacitance of a single-pixel was found to be 13.7 fF. The PDE of the MAPD-3NM-2 type photodiode varied between 16% and 35%, depending on the applied voltage.

**Fig. 3:**
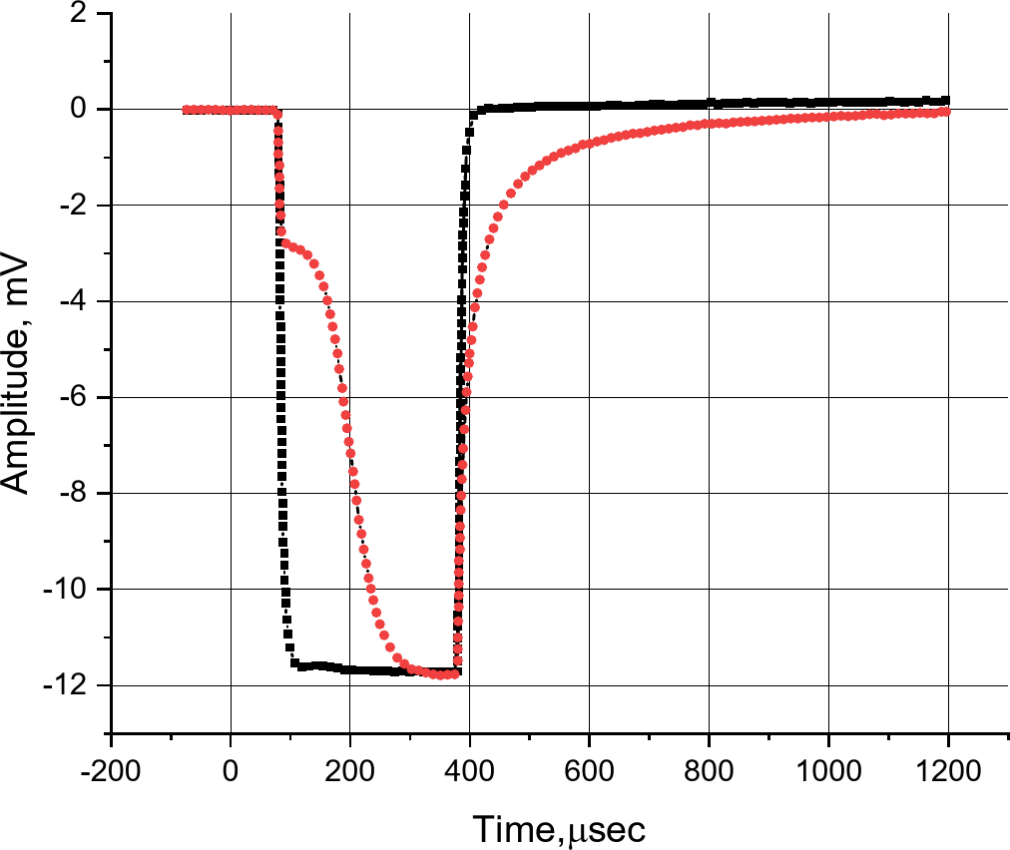
Waveforms of the photo-response of the PIN and MAPD-3NM-2 type at 450 nm wavelengths were recorded with a standard signal OWON Smart DS8202 oscilloscope.

In MAPD-3NM-2 type photodiodes, the quenching of the avalanche process occurs due to electrons collected in the potential well where the pixels are located. To determine the number of these electrons, a rectangular light pulse with a width of 250 microseconds and a frequency of 5 kHz was applied to the MAPD-3NM-2 type photodiode using an LED. Additionally, a PIN photodiode was used to calculate the number of photoelectrons generated by a rectangular LED pulse. The signal from the MAPD-3NM-2 type photodiodes was fed to an oscilloscope through a 5 kΩ load resistor. Figure 3 shows the pulse shapes for the PIN (black) and MAPD-3NM-2 type photodiodes (red). Using the difference in the areas of both signals, the area captured by the MAPD-3NM-2 type photodiode pixels was found to be 9.38 × 10^− 7^V×sec. Dividing this value by the load resistance (5 kΩ) gave the total charge captured by the pixels as 186.6 × 10^− 12^ C. Dividing this charge by the total number of pixels (3.7 × 3.7 × 4857 = 66,500 pixels), the charge per pixel was determined to be Q_pix_ = 186.6 × 10^− 12^ C / 66,500 = 2.81 × 10^− 15^ C. Dividing this charge by the electron charge, the number of electrons per pixel was found to be 1.75 × 10^4^ electrons. This charge accumulates in each pixel, ensuring the reduction of the electric field and resulting in the quenching of the avalanche process.

**Fig. 4:**
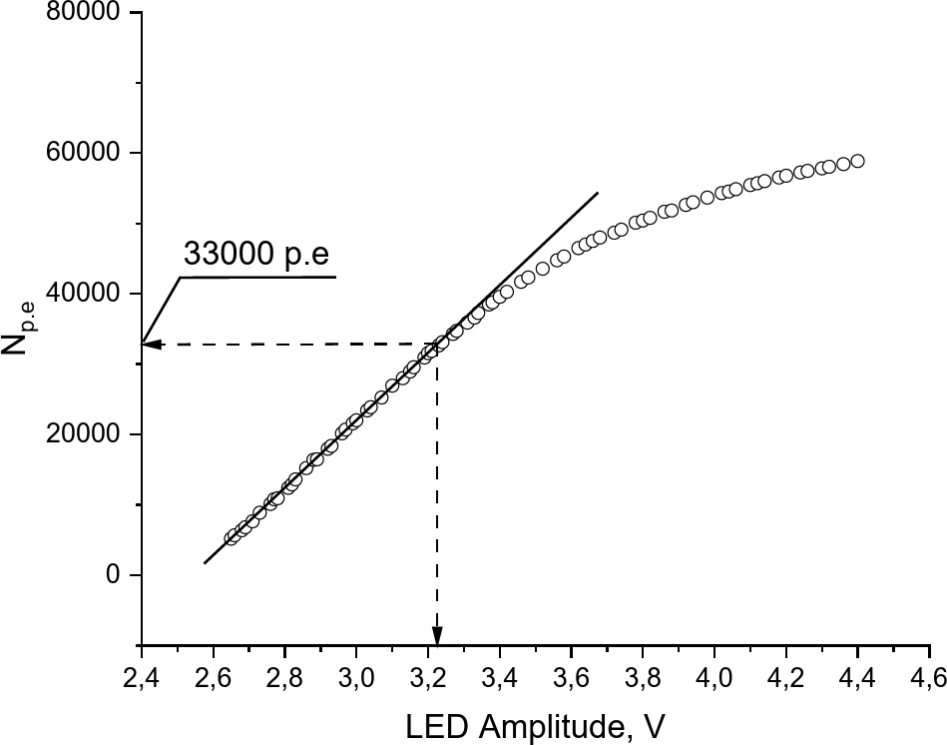
The dependence of the number of photoelectrons detected by the MAPD-3NM-2 type photodiode on the amplitude of the LED.

A 450 nm LED light-emitting diode was used to determine the linearity of the MAPD-3NM-2 type photodiode. The frequency of the light pulse from the generator to the LED was 3 kHz, with a pulse width of 40 ns. By increasing the signal amplitude from 2.6 to 4.6 V, the photo, and dark currents of the MAPD-3NM-2 type photodiode were measured, and then the number of photoelectrons per pulse was calculated. Figure 4 shows the dependence of the number of photoelectrons per pulse on the signal amplitude applied to the LED as recorded by the MAPD-3NM-2 type photodiode. As seen from the dependence, the MAPD-3NM-2 type photodiode maintained its linearity even when the number of photoelectrons per pulse reached 33.000.

**Fig. 5:**
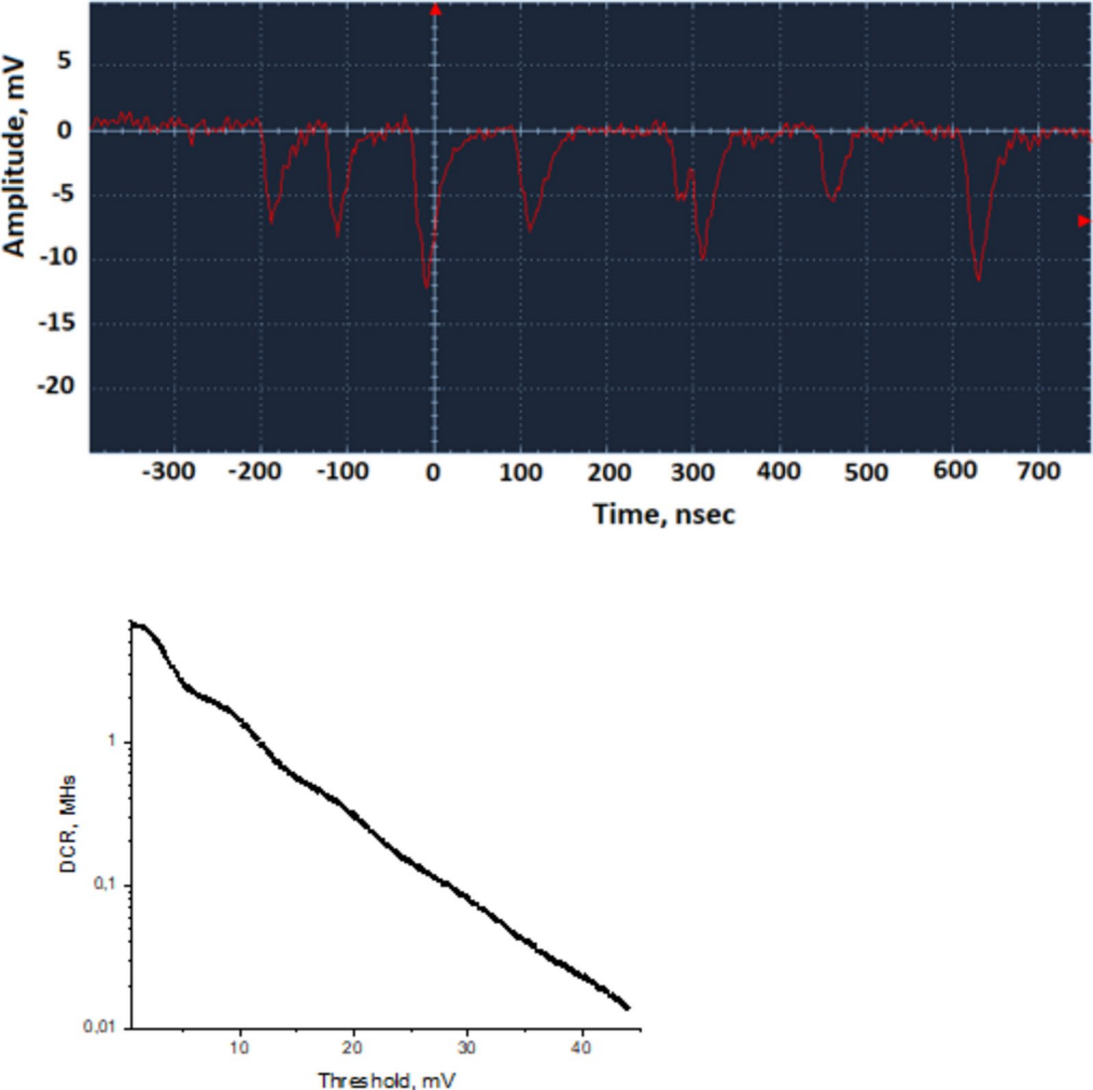
The signal shape of dark electrons in the MAPD-3NM-2 type photodiode was recorded with the OWON Smart DS8202 oscilloscope and variation of DCR for the threshold value.

To determine the dark count rate (DCR) of the MAPD-3NM-2 type photodiodes, a CAEN amplifier with a gain of 150 was used. The bandwidth of this type of amplifier was 200 MHz. A voltage of 55 V (dark current 600 nA) was applied to the MAPD-3NM-2 type photodiode, and the resulting dark counts were recorded using an OWON Smart DS8202 oscilloscope (Bandwidth-200 MHz) by varying the threshold amplitude. Figure 5 shows the signal shapes corresponding to one and two electrons on the oscilloscope screen. The amplitude of the dark electron corresponding to one electron was approximately 5.4 mV. The DCR experiment was conducted at a temperature of 22 °C. Figure 5 shows the variation of the DCR as a function of the threshold amplitude. The DCR varied from 0.01 to 5 MHz depending on the threshold value. The DCR corresponding to one electron was 2.3 MHz. The DCR per 1 mm² was calculated as DCR (1.e) = 2.3 MHz / (3.7 × 3.7 mm²) = 168 kHz / mm². The approximate DCR per pixel was DCR (1 pixel) = 168 kHz / 4857 pixels = 34 Hz/pixel.

**Fig. 6:**
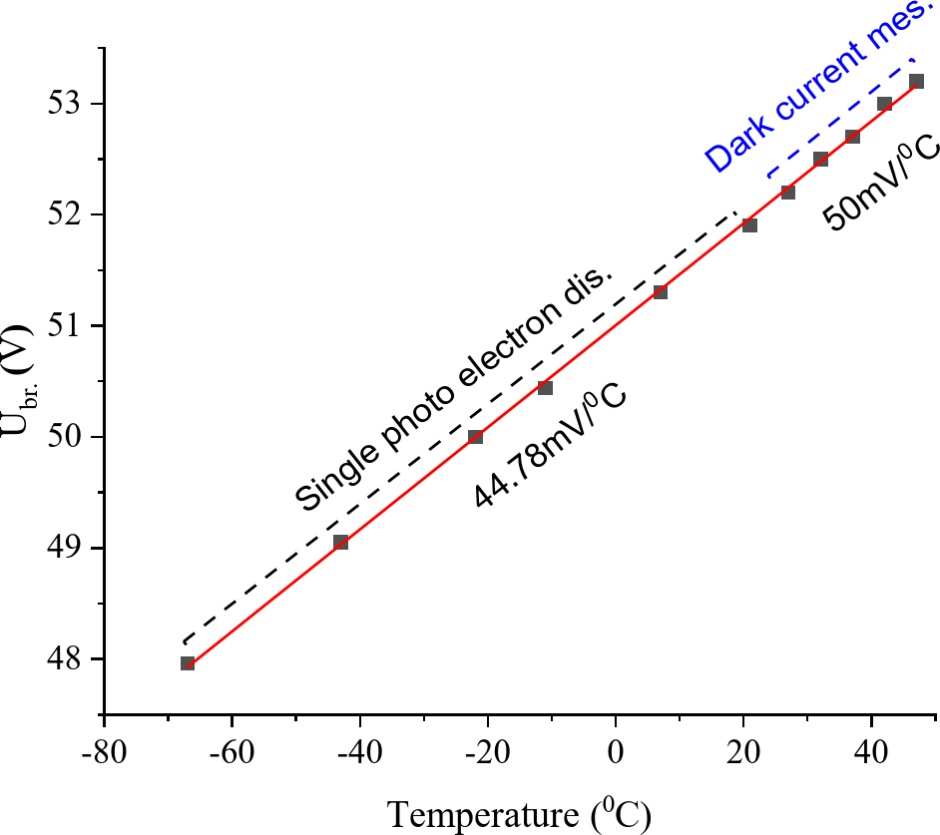
Dependence of the breakdown voltage of the MAPD-3NM-2 type photodiode on temperature.

Figure 6 shows the dependence of the breakdown voltage of the MAPD-3NM-2 type photodiode on temperature. Low-temperature experiments (−67 to + 7 °C) were conducted using liquid nitrogen. During the determination of the breakdown voltage in the temperature range of −67 to + 21 °C, the single photoelectron distribution method was used. It was determined that the breakdown voltage changed by (44.78 ± 0.31) mV/°C as a function of the temperature. At higher temperatures, the single photoelectron distribution method could not be used to determine the breakdown voltage due to a sharp increase in dark current, which triggers the avalanche process in the pixels and makes it impossible to obtain the single photoelectron distribution. Therefore, in the temperature range of + 27 to + 47 °C, the breakdown voltage was determined using the differential dependence of the dark current. It was found that the breakdown voltage changed by (50 ± 2) mV/°C as a function of the in this range. Overall, in the temperature range of −7 to + 47 °C, the breakdown voltage changed according to the following relationship: U_br_.=(51.003 ± 0.0014)+(45.92 ± 0.38)mV×T( °C). It was determined that the breakdown voltage changes by 45.9 mV/°C with temperature.

**Fig. 7:**
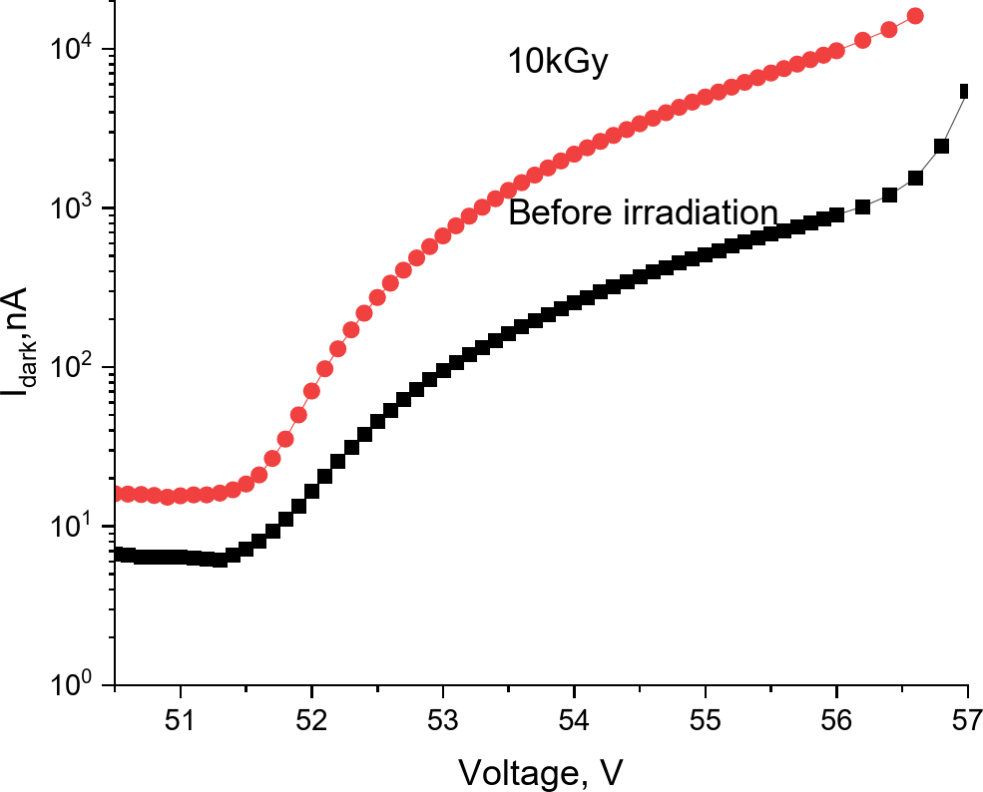
Dependence of the dark current of the MAPD-3NM-2 type photodiode on voltage before and after gamma irradiation.

In Fig. 7, the effect of gamma radiation on the physical properties of the MAPD-3NM-2 is investigated. Cobalt-60 with an activity of 131 GBq was used as the gamma radiation source. The MAPD-3NM-2 type photodiode was irradiated with a total dose of 10 kGy in one run. This irradiation dose is considered equivalent to the dose detectors receive in 10 years of cosmic research^52–54^. The initial dark current of the MAPD-3NM-2 type photodiode at operating voltage was 510 nA.After irradiation with a gamma dose of 10 kGy, the dark current of the photodiode increased to 4967 nA, is an increase by a factor of 9.7. Such an increase in dark current is attributed to the emergence of new generation centers due to radiation damage.

**Fig. 8:**
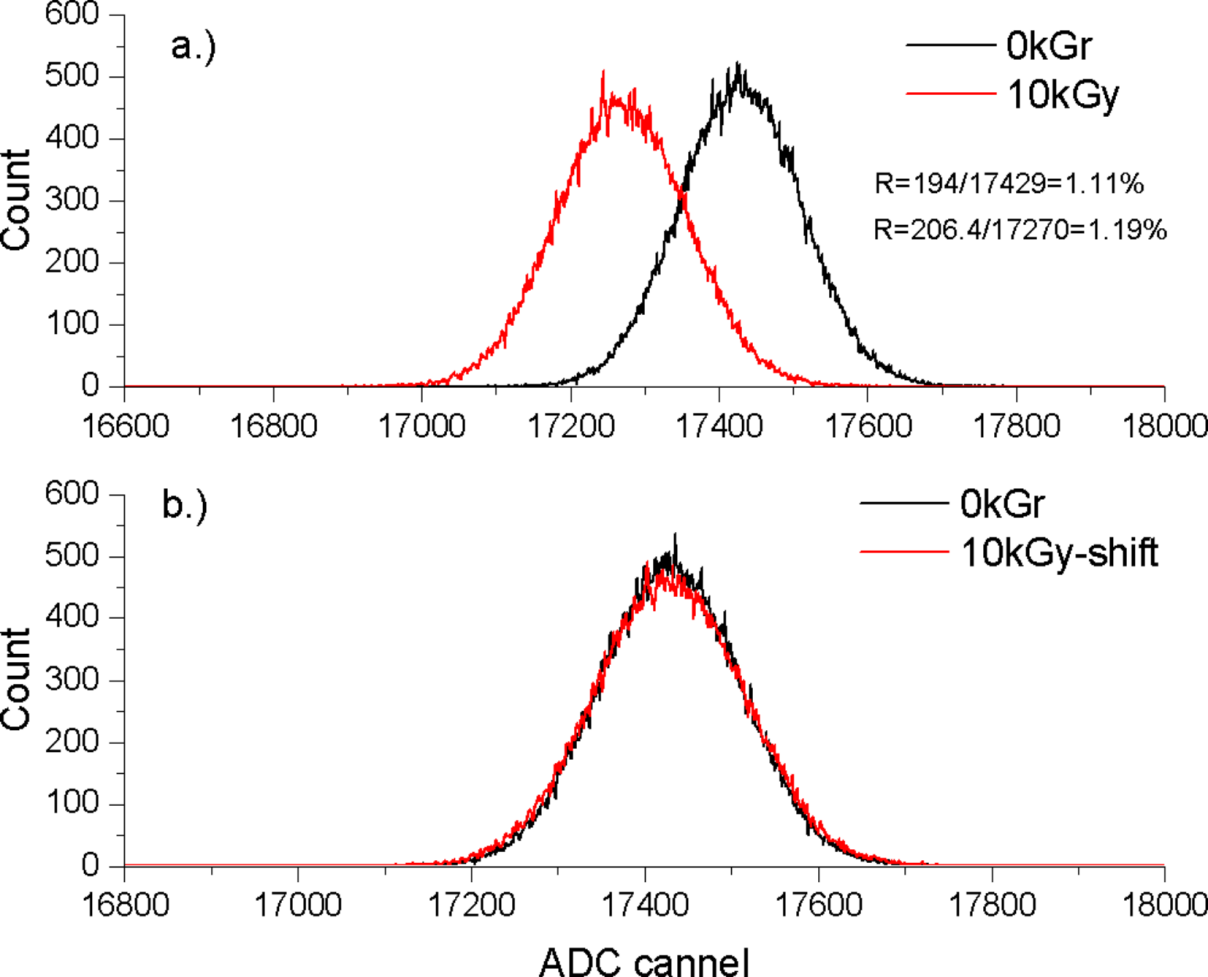
Amplitude distribution of the photo-signals recorded with the MAPD-3NM-2 type photodiode before and after exposure to gamma radiation with dose of 10 kGy: (**a**) as measured, and (**b**) with peak maxima positions normalized to overlap the peaks for a comparison of their widths.

In Fig. 8a, the sensitivity of the MAPD-3NM-2 type photodiode to a 450 nm LED is shown to vary with dose. Rectangular pulses with a duration of 100 ns, an amplitude of 3.1 V, and a frequency of 5 kHz were applied to the LED from the generator. The signal from the MAPD-3NM-2 type photodiode was analysed using a CAEN-5720 ADC. The amplitude and FWHM of the recorded photo signal were calculated using the Gaussian distribution. A PIN photodiode was used to determine the number of photons per pulse packet. At this time, a total of 8000 photons were detected per pulse packet. Before irradiation, the amplitude of the photo-signal recorded with the MAPD-3NM-2 type photodiode at an overvoltage of 3 V fell to channel 17,429 of the CAEN-5720 ADC, with an FWHM of 194.05 channels. The peak resolution for the recorded photo signal with the MAPD-3NM-2 type photodiode was 1.11%. After irradiation with a gamma dose of 10 kGy, the amplitude of the photo-signal corresponding to the same photon shower fell to channel 17,270 of the ADC, with an FWHM of 206.4 channels. The peak resolution for the recorded photo signal with the MAPD-3NM-2 type photodiode was 1.19%. After irradiation, the change in the amplitude of the photo signal was 0.91%, and the change in the FWHM was 6.3%. The peak resolution decreased by ~ 7%.

In Fig. 8b, the differences in FWHM are visually shown by plotting the two photo signals normalized to the same peak position. The change in peak resolution is explained by the triggering of avalanche processes of charge carriers generated by radiation defects in the pixels (in other words, that the pixel is busy or that the avalanche process in the pixel is not completely quenched). These resulted in different amplitudes of the photo-electron recorded in the same pixel compared to the pixel when the avalanche process was completely quenched (or the avalanche process was not triggered by the carrier). Furthermore, due to defects, the increased dark current also adds different charges to the recorded photo signal, which in turn contributes to the expansion of the FWHM corresponding to the photo signal^54^.

Thus, investigations into gamma irradiation have shown that even after a dose of 10 kGy, the photodiode largely retains its operational parameters.The main parameters of MAPD-3NM-2 photodiode is given Table 1.

**Table 1:** The main parameters of the MAPD-3NM-2 type photodiode.

Parameters	MAPD-3NM-2
Active area (mm)	3.7 × 3.7
Pixel diameter/pitch (um)	12/15
Pixel density (pixels/mm^2^)	4857
Breakdown voltage	52.3
Operation voltage	53–56.5
Photon detection efficiency (%)	~ 35 (450–550 nm)
Dark current (nA)	500
Gain	1–3 × 10^5^
DCR (1 e) per pixel (Hs)	34
Pixel capacitance (fF)	13.7
Diode capacitance(pF)	157
Number of electrons per pixel for quenching avalanche proses (electrons).	1.75 × 10^4^
Linearity range (p.e)	33,000
Temperature coefficient of breakdown voltage (mV/^0^C)	45.9
Radiation resistance (Change in amplitude resolution − 10 kGy)	< 7.5%

### Parameters of scintillators

LSO and LaBr3(Ce) are two in-organic scintillators that have been used in the measurements. The dimensions and parameters of these scintillators used are given in Table 2.

**Table 2:** Main parameters of LSO and LaBr_3_(ce) scintillators^27,55,56^.

Parameters	LSO	LaBr_3_(Ce)
Density (g/cm^3^)	7.4	5.1
Effective atomic number	75	46.9
Light output (photons/MeV)	30,000	60,000
Maximum wavelength (nm)	420	380
Dimensions (mm)	15 × 15 × 50	15 × 15 × 50
Internal radiation background	β (182 keV 593 keV) γ (55.66 keV, 63.2 keV, 88.75 keV, 202.33 keV, 307.17 keV, 401.13 keV, 509 keV)	β (263 ÷ 1400 keV) γ (32 keV, 789 keV, 1436 keV) α (5 ÷ 7.4 MeV)
Decay time (ns)	40	16
Hygroscopic	No	Yes

**Fig. 9:**
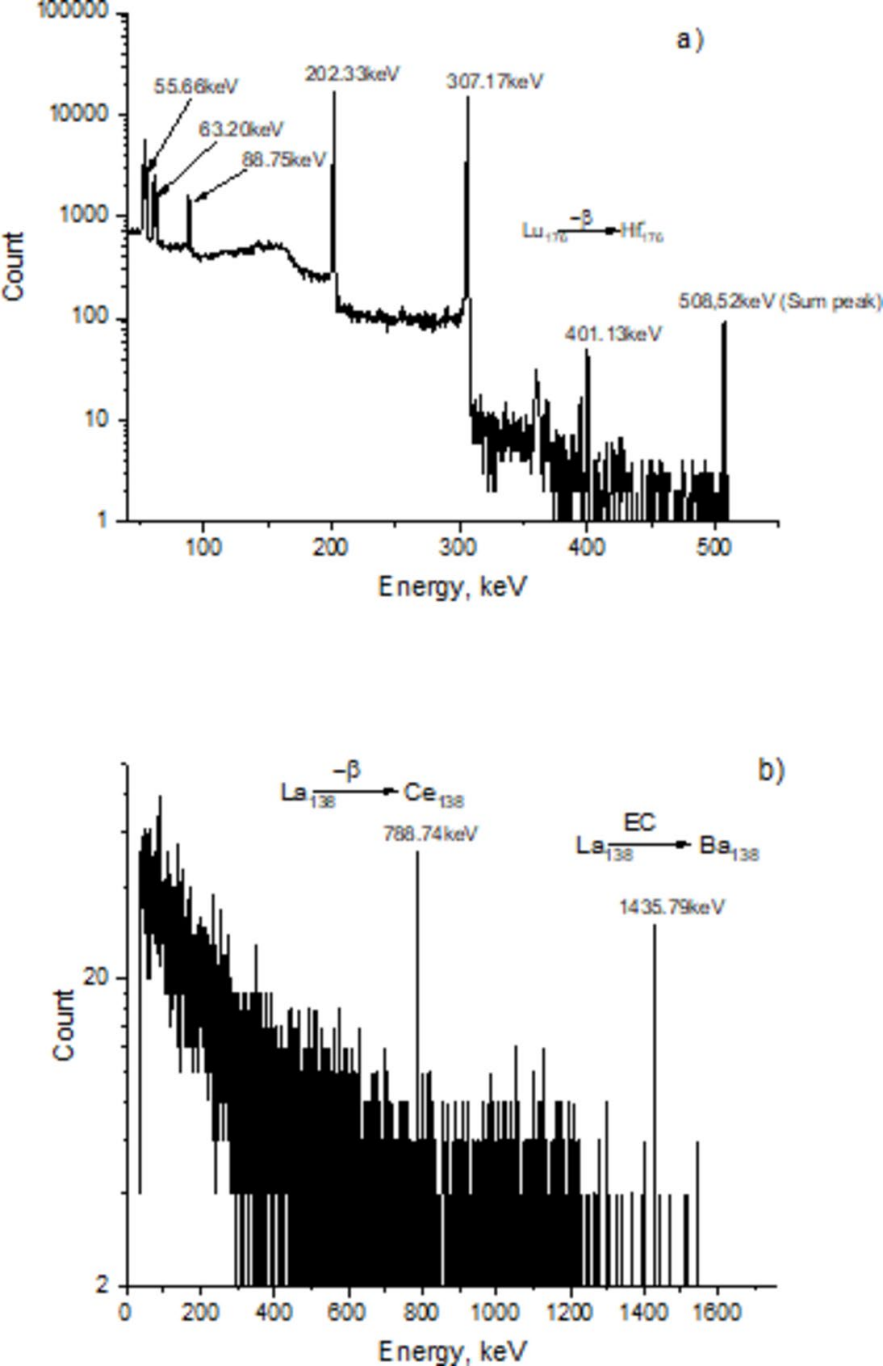
Internal background captured with an HPGe detector for LSO (**a**) and LaBr_3_(Ce) (**b**) scintillators.

In Fig. 9, the internal background of the scintillators used in the experiment is shown, captured with an HPGe detector. A measurement duration of 5721 s was selected. As seen from the spectrum, gamma rays with energies of 55.66 keV, 63.2 keV, 88.75 keV, 202.33 keV, 307.17 keV, 401.13 keV, and 508.52 keV are observed in the LSO scintillator. These characteristic gamma rays are created by the transition of the radioactive Lu-176 isotope inside the scintillator to the excited states of Hf-176 through β-decay. The energies of these emitted gamma rays were 55.66 keV, 63.2 keV, 88.75 keV (B.R-0.141), 202.33 keV (B.R-0.91), 307.17 keV (B.R-0.936), and 401.13 keV (B.R -0.0035). The peak corresponding to the energy of 508.52 keV in the spectrum is formed due to the simultaneous detection of two gamma rays with energies of 202.33 keV and 307.17 keV. The determined specific activity of the Lu-176 isotope in the LSO scintillator was 1400 ± 100 Bq/g.

The internal background captured with an HPGe detector for the LaBr_3_(Ce) scintillator over 5641 s is shown in Fig. 8. Two gamma lines with energies of 788.74 keV and 1435.79 keV were observed in the spectrum. The 788.74 keV gamma line is emitted when the ^138^La isotope transitions to the ^138^Ce isotope through beta decay. The other gamma line occurs when ^138^La transitions to ^138^Ba- isotope through electron capture (EC). The specific activity of ^136^La isotope in the LaBr_3_(Ce) scintillator was 3.4 ± 0.2 Bq/g.

### Testing of a scintillator detector based on the MAPD-3NM-2 type photodiode

**Fig. 10:**
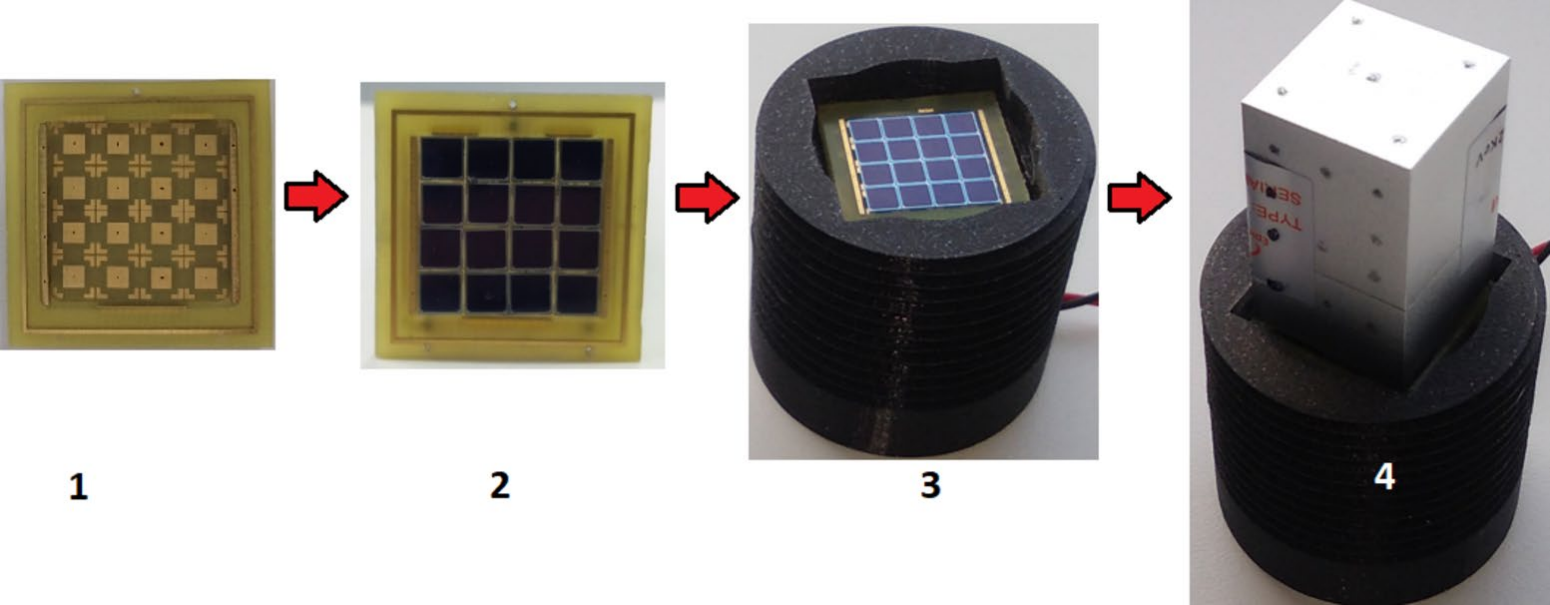
illustrates the configuration of the array used in the experiment. The array consisted of 16 elements of MAPD-3NM-2 type photodiodes. The total area of the array was 289 mm^2^, with an active area of 219.04 mm^2^. The fill factor of the array was approximately 76%.

Figure 10. Illustrates the configuration and top view of the 16-element MAPD-array based on MAPD-3NM-2 type photodiodes: 1 shows the array housing, 2 shows the array assembled with MAPD-2 type photodiodes, 3 displays the base constructed with the MAPD-array using a 3D printer, and 4 depicts the MAPD array and scintillator.

**Fig. 11:**
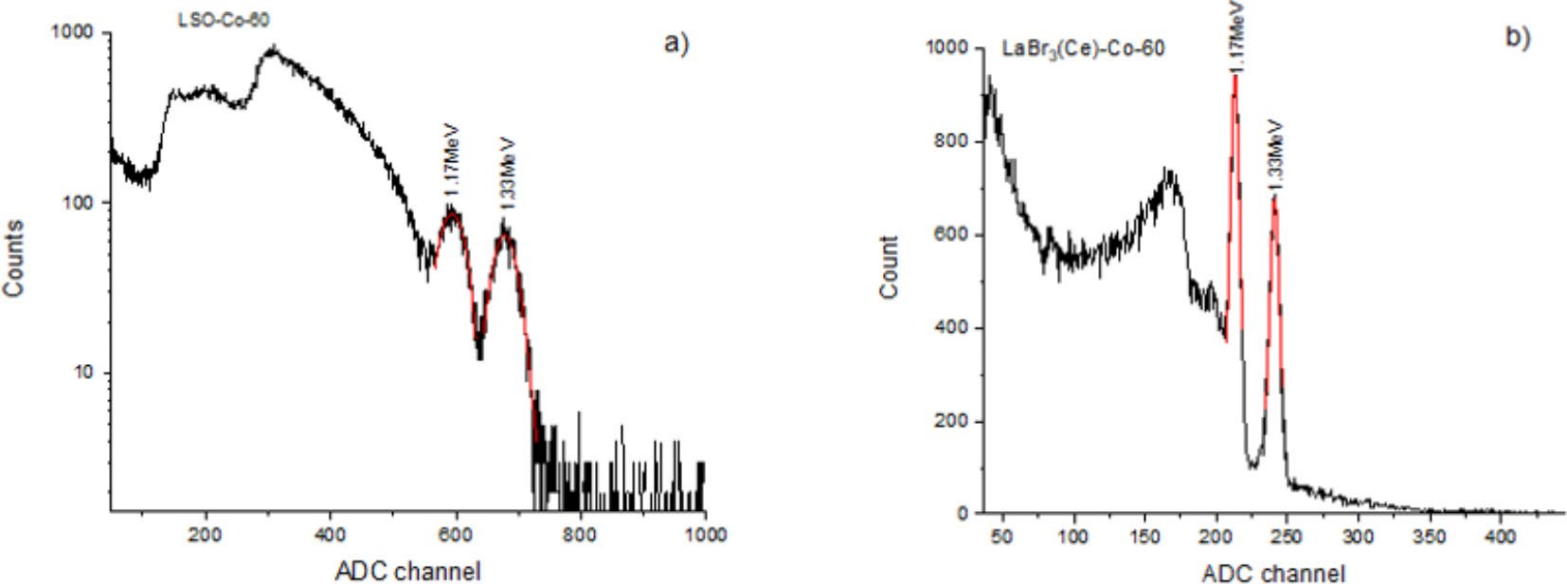
The spectra of gamma rays from the ^60^Co source captured with the (a) LSO and (b) LaBr_3_(Ce) scintillator coupled to MAPD array.

When measuring with Co-60 and AmBe sources, no lead shielding was used on the detectors. In Fig. 11, the spectrum captured with the MAPD-matrix and LSO scintillator for the ^60^Co source is shown. In this experiment, a variable gain of 23 dB and an integration window of 140 nanoseconds were selected for the MAPD Spectrig.The detailed information about MAPD Spectrig is given reference^39^.The energy resolution for the 1.17 MeV and 1.33 MeV gamma rays emitted by the ^60^Co source was 10.7% and 7%, respectively. It should be noted that the poorer energy resolution for the 1.17 MeV gamma ray compared to the 1.33 MeV gamma ray was primarily due to the influence of the Compton continuum of the 1.33 MeV gamma ray. Therefore, the 1.33 MeV gamma ray from the source was used to compare the energy resolution obtained with both scintillators.

Figure 11 also displays the spectrum captured with the LaBr_3_(Ce) scintillator for the ^60^Co source. In this case, a variable gain of 10 dB and an integration window of 135 nanoseconds were selected for the MAPD Spectrig. The energy resolution for the 1.17 MeV and 1.33 MeV gamma rays emitted by the ^60^Co source was 3.25% and 3%, respectively. Thus, the energy resolution obtained with the LaBr3(Ce) scintillator for the 1.33 MeV gamma ray was 2.5 times better compared to LSO. The difference in energy resolution is proportional to the light output of the scintillators and their maximum emitted wavelength.

**Fig. 12:**
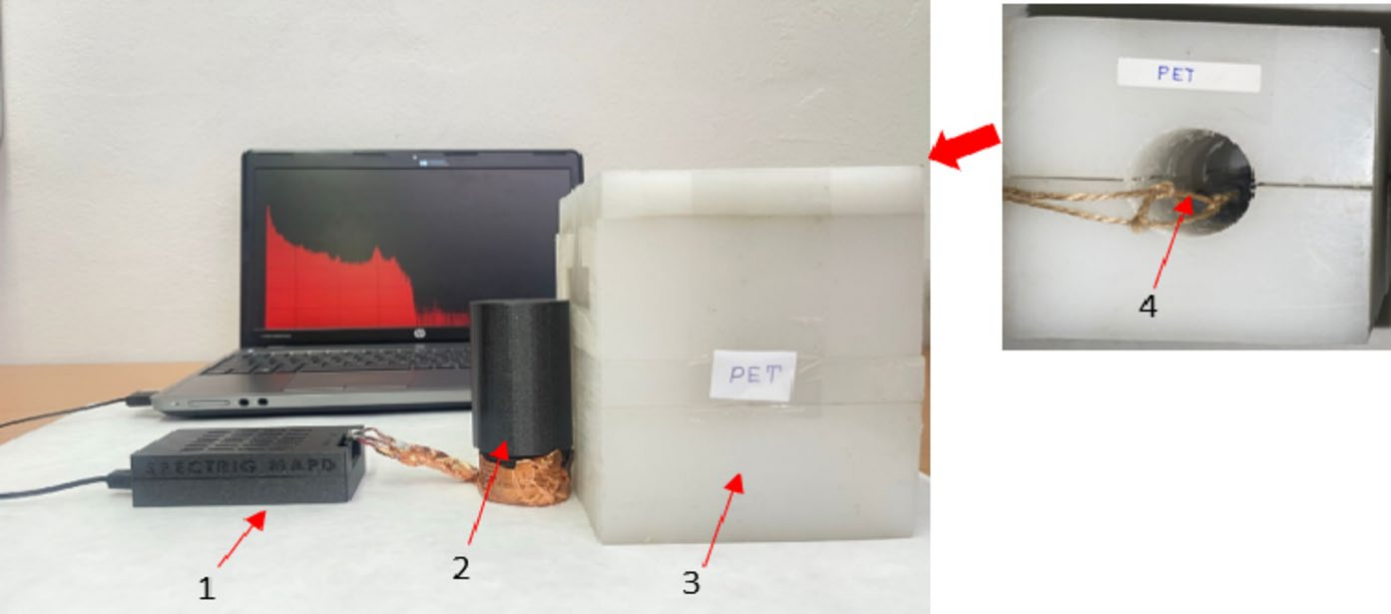
Experimental setup for measurement. 1 - SPECTRIG MAPD, 2 – MAPD array assembled with LaBr_3_(Ce) or LSO scintillator, 3 - PET target, and 4 - AmBe neutron source.

Figure 12 illustrates the experimental setup used with the AmBe source. The activity of the AmBe source used was 37 GBq. The neutron source was placed inside a PET-containing box measuring 12 cm × 12 cm × 15.5 cm. At this time, the average energy of the fast neutrons emitted from the source was 4 MeV. Additionally, when a neutron interacts, the excited carbon atom resulting from the reaction emits a gamma ray with an energy of 4.44 MeV: ^9^Be + α → ^13^C* →^12^C + n + γ. In the PET composition, these neutrons participate in elastic and inelastic scattering with hydrogen and carbon. When fast neutrons undergo inelastic scattering with a carbon atom, they transfer part of their energy to the carbon atom’s nucleus. As a result, the nucleus becomes excited and emits a gamma ray with an additional energy of 4.44 MeV: n + ^12^C → ^12^C* + n’ → ^12^C + γ (4.44 MeV). These events ensure the observation of events corresponding to gamma rays with an energy of 4.44 MeV in the spectrum. The fast neutrons emitted from the source participate in numerous elastic scattering events with hydrogen, losing their energy and being converted into thermal neutrons. These thermal neutrons are then captured by hydrogen nuclei to form deuterium (H^2^_1_) isotopes, resulting in the emission of a gamma ray with an energy of 2.223 MeV: n_1_ + H^1^_1_ → H^2^_1_ + γ (2.223 MeV).

**Fig. 13:**
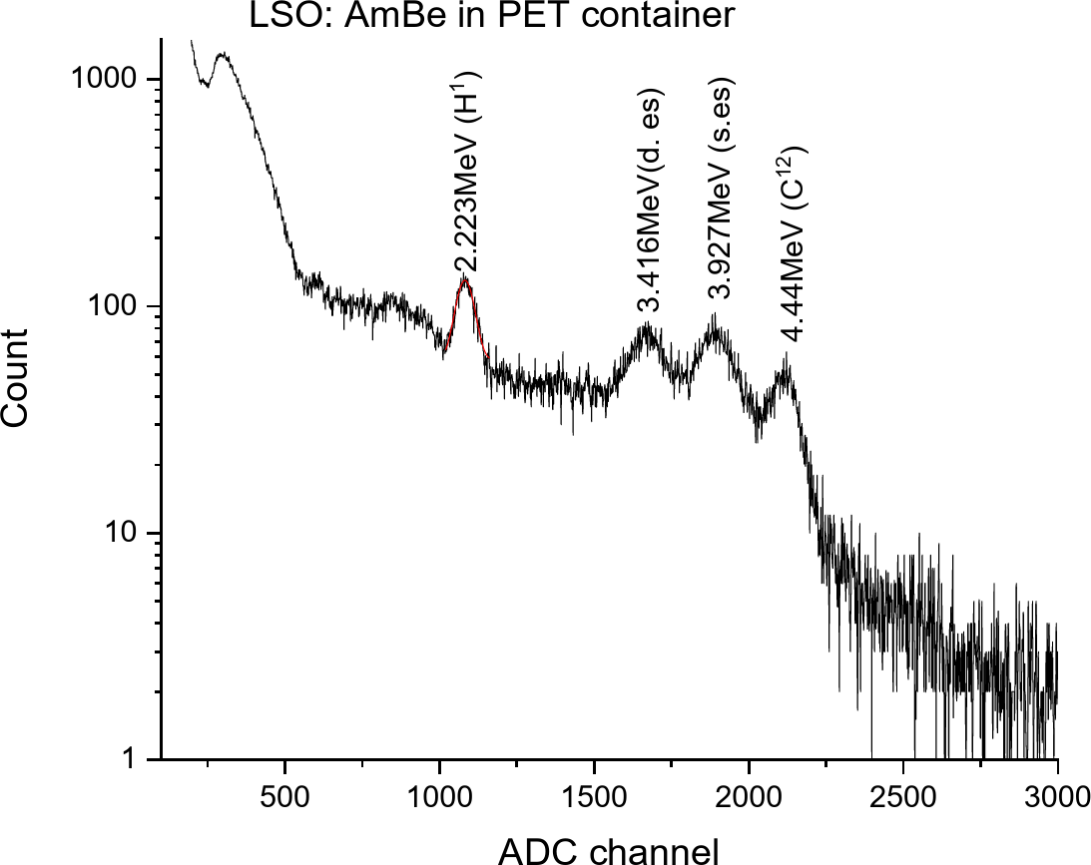
Spectrum of gamma rays emitted from the AmBe source and PET target with the LSO + MAPD array.

In Fig. 13, the spectrum recorded with the LSO scintillator is shown. An 8-minute measurement duration was chosen for both scintillators. As seen from the spectrum, alongside 2.223 MeV energy gamma rays, 4.44 MeV energy gamma rays are also observed. The high density of the LSO scintillator allows for high detection efficiency of gamma rays. As seen from the spectrum, the LSO scintillator allows for the detection of gamma rays at the upper energy range of the spectrum: 4.44 MeV, 3.927 MeV (4.44–0.511 MeV) (Single escape peak), and 3.416 MeV (4.44 MeV − 1.022 MeV) (Double escape peak). The energy resolution for the 2.223 MeV energy gamma ray with the LSO-based detector was 6.89%. The area of the photopeak corresponding to the 2.223 MeV energy gamma ray was 4814 events. It was determined using a Gaussian fit.

**Fig. 14:**
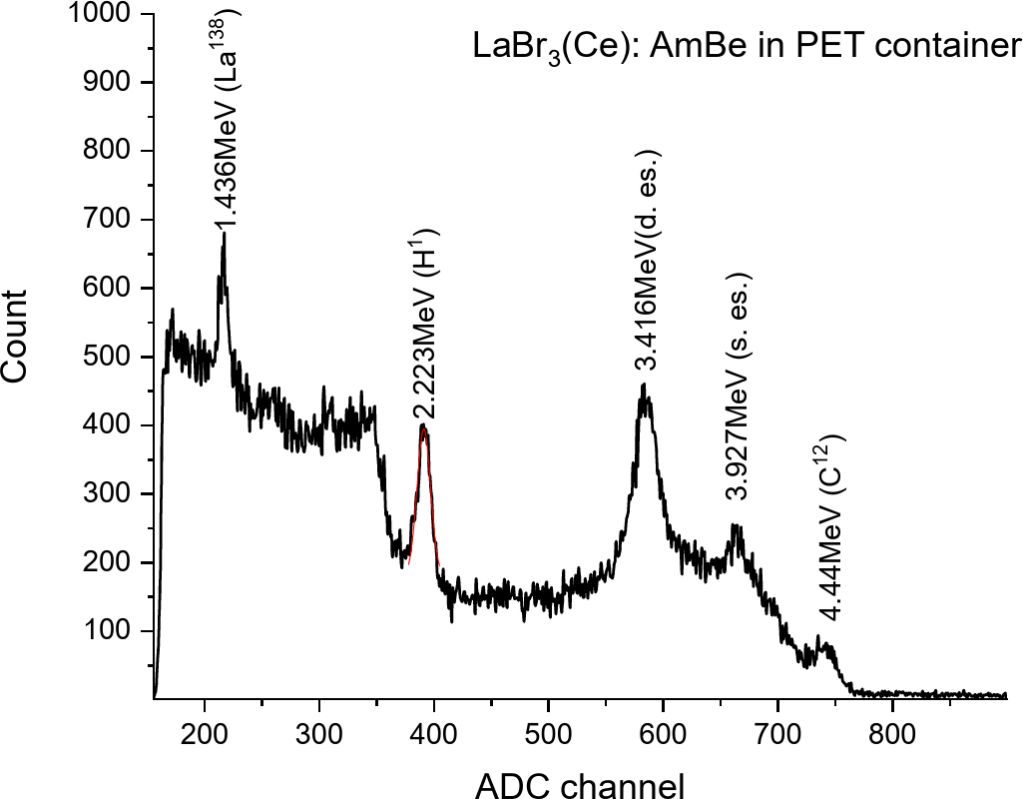
Spectrum of gamma rays emitted from the AmBe source and PET target with the LaBr_3_(Ce) + MAPD array.

In Fig. 14, the spectrum recorded with the LaBr_3_(Ce) scintillator is shown. As seen from the spectrum, alongside 2.223 MeV energy gamma rays, 4.44 MeV energy gamma rays are also observed. Additionally, a 1436 keV energy gamma ray resulting from La-138 decay is observed in the spectrum. This gamma line was also observed when measuring the intrinsic background of LaBr3(Ce) using HPGe (Fig. 9b). As seen from the spectrum, the LaBr_3_(Ce) scintillator allows for the detection of peaks corresponding to gamma rays at the upper energy range of the spectrum: 4.44 MeV, 3.927 MeV (4.44–0.511 MeV), and 3.416 MeV (4.44 MeV − 1.022 MeV). It should also be noted that the internal radioactive contamination due to ^227^Ac and the events created by alpha particles (5-7.4 MeV) emitted by its daughter nuclei cover the energy range of 1.8–2.5 MeV in the spectrum^28,56^. These events affect the spectrum of the 2.223 MeV energy gamma ray. The energy resolution for the 2.223 MeV energy gamma ray with the LaBr_3_(Ce)-based detector was 3.55%. The area of the photopeak corresponding to the 2.223 MeV energy gamma-ray in this spectrum was 3960 events.

### Neutron simulation

**Fig. 15:**
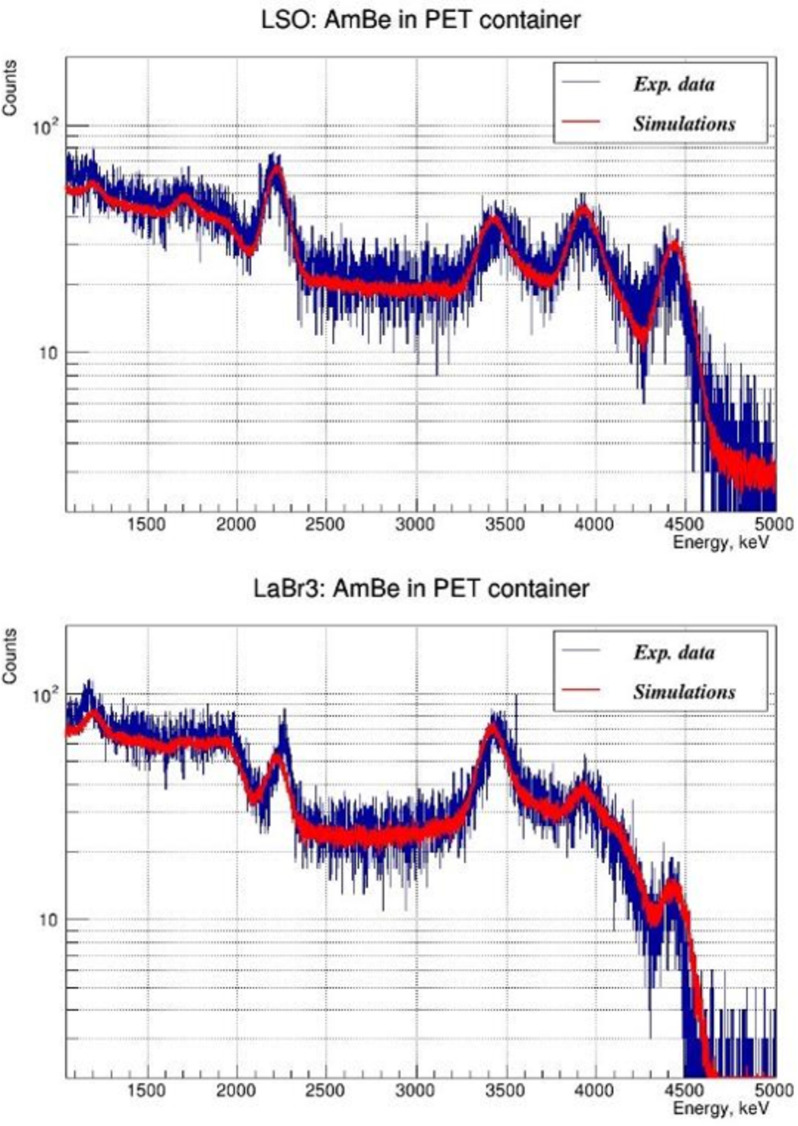
Comparison of simulated and experimental spectra of AmBe(PET) for LSO (top) and LaBr3(Ce) (bottom).

To verify the measurements made with the neutron source, the latter was simulated using Geant4 framework^57^ (version 4-10-06) with the physics list QGSP_BIC_HP, which performs precision simulations of neutrons in matter. The scintillator and source geometry shown in Fig. 12 were used. The AmBe source was simulated as a mixture of three components: fast neutrons, slow neutrons, and 4.44 MeV gamma rays accompanying the emission of a neutron from the AmBe source in 75% of cases (due to the alpha reaction on beryllium). Finally, the simulated spectrum was broadened to the resolution estimated from the experimental data.The simulation results are shown in Fig. 15. It shows that the simulated spectra are in good agreement with experimental data in the energy range (1–5) MeV.

**Fig. 16:**
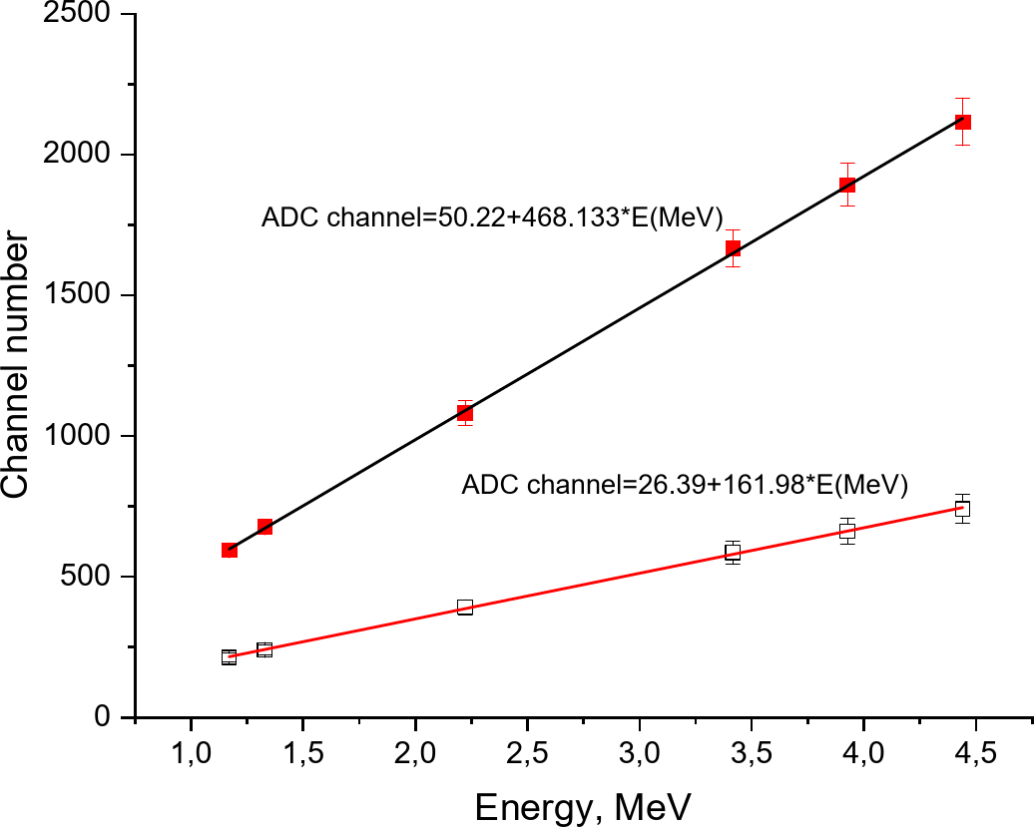
Calibration curve of the detector based on LaBr_3_(Ce) and LSO + MAPD array.

The calibration curve obtained for both scintillators is shown (Fig. 16). The MAPD array maintained its linearity with both scintillators and the following linear dependencies between the ADC channel and energy were determined:

For LSO ADC channel = 50.22 + 468.133 × E(MeV).

For LaBr_3_(Ce) ADC channel = 26.39 + 161.98 × E(MeV).

Thus, gamma rays resulting from the hydrogen interactions were recorded with both scintillators. Gamma rays arising specifically from ^12^C, and their Compton scattering add complexity to the spectrum and negatively impact the energy resolution of the detectors. Most gamma rays from ^12^C in the spectrum are formed through the reaction ^9^Be + α → ^13^C* →^12^C + n + γ, especially in the highly active AmBe source. The high density of the LSO scintillator allows for 21.5% more efficient detection of 2.223 MeV gamma rays compared to the LaBr_3_(Ce) scintillator. The best energy resolution for the 2.223 MeV gamma ray associated with hydrogen was 3.55% with the LaBr_3_(Ce) scintillator. This energy resolution obtained with the LaBr_3_(Ce) scintillator is 48.5% better compared to the LSO scintillator. The worse energy resolution in comparison with LSO is due to the higher light output of LaBr_3_(Ce). The calibration curve for detectors based on MAPD array and LSO-LaBr_3_(Ce) scintillators was linear in the energy range of 1.1–4.44 MeV.

The experiments conducted demonstrated that both detectors can provide information about the presence of hydrogen in the target.

Future experiments are planned to improve the obtained results:

- To eliminate the negative impact of gamma rays from ^12^C and their Compton scattering on the gamma spectrum of hydrogen, experiments will be conducted using a monoenergetic neutron generator.
- Use of CeBr scintillators with low internal background.
- Investigation of changes in the number of gamma events corresponding to hydrogen (peak area) recorded by the detector using various hydrogen-containing materials (water-ice, etc.) and volumes.
- Testing the detector in various combinations (soil-stone-water) is planned.
- Reducing the influence of background radiation in the spectrum using lead shielding.

## Conclusion

The ongoing research on the gamma ray spectrometer based on new MAPD-3NM-2 type photodiodes with LaBr_3_(Ce) and LSO scintillators has demonstrated promising results for hydrogen detection. The comparative analysis between the two scintillators indicates that the LaBr_3_(Ce) scintillator provides superior energy resolution for the 2.223 MeV gamma-ray, associated with hydrogen, compared to the LSO scintillator. Specifically, the LaBr_3_(Ce) scintillator achieved an energy resolution of 3.55%, which is 48.5% better than the 6.89% resolution achieved with the LSO scintillator. However, the LSO scintillator-based detector detected 21.5% more 2.223 MeV gamma rays, indicating a trade-off between detection efficiency and energy resolution.

These findings suggest that both scintillators have distinct advantages depending on the specific requirements of the detection task. The LaBr_3_(Ce) scintillator, with its better energy resolution, is particularly advantageous for precise gamma-ray spectroscopy. In contrast, the LSO scintillator, with its higher detection efficiency, may be better suited for scenarios where maximizing the count of detected gamma rays is critical.

The research also highlights the robustness of MAPD-based detectors in space research applications, given their compact size, low power consumption, and resistance to radiation. This ongoing study contributes valuable insights into optimizing detector configurations for planetary exploration and other applications requiring precise and efficient gamma-ray detection.

## Data Availability

All data generated or analysed during this study are included in this published article.
